# Temperature Hysteresis Calibration Method of MEMS Accelerometer

**DOI:** 10.3390/s25196131

**Published:** 2025-10-03

**Authors:** Hak Ju Kim, Hyoung Kyoon Jung

**Affiliations:** 1 Advanced Development Team, Microinfinity Co., Ltd., Suwon 16229, Republic of Korea; hzkim@minfinity.com; 2 Sensor Advanced Development Center, Microinfinity Co., Ltd., Suwon 16229, Republic of Korea

**Keywords:** MEMS accelerometer, temperature hysteresis, calibration, thermal drift, navigation sensors

## Abstract

Micro-electromechanical system (MEMS) sensors are widely used in various navigation applications because of their cost-effectiveness, low power consumption, and compact size. However, their performance is often degraded by temperature hysteresis, which arises from internal temperature gradients. This paper presents a calibration method that corrects temperature hysteresis without requiring any additional hardware or modifications to the existing MEMS sensor design. By analyzing the correlation between the external temperature change rate and hysteresis errors, a mathematical calibration model is derived. The method is experimentally validated on MEMS accelerometers, with results showing an up to 63% reduction in hysteresis errors. We further evaluate bias repeatability, scale factor repeatability, nonlinearity, and Allan variance to assess the broader impacts of the calibration. Although minor trade-offs in noise characteristics are observed, the overall hysteresis performance is substantially improved. The proposed approach offers a practical and efficient solution for enhancing MEMS sensor accuracy in dynamic thermal environments.

## 1. Introduction

Micro-electromechanical system (MEMS)-based accelerometers or gyroscopes are utilized in various navigation applications due to their advantages of low cost, low power consumption, and compact size [1,2]. In general, the required sensor performance varies depending on the navigation performance demanded by each application. Therefore, calibration mechanisms are implemented to enhance sensor performance and provide accurate sensor data to the navigation system [3,4,5]. For MEMS accelerometers, sensor calibration typically addresses misalignment, scale factors, nonlinearity, white noise, and temperature drift [6]. Compared with conventional mechanical sensors, MEMS devices have lower thermal capacity and stronger temperature dependence, making them more susceptible to performance degradation under varying thermal conditions [7,8].

Temperature drift can often be compensated with models based on external temperature sensors mounted outside the MEMS package. However, hysteresis effects caused by internal temperature gradients, fabrication variations, and sensor placement remain difficult to correct with conventional methods alone. With the growing demand for navigation-grade performance, temperature hysteresis has become a critical performance parameter [9].

To mitigate temperature hysteresis, many previous studies have been conducted. Micro-oven control has been used to stabilize internal temperature [10,11], while integrated temperature sensors such as platinum films or resonators have been incorporated into MEMS devices to improve thermal tracking [12,13]. Other studies focus on materials, components, or structural designs that reduce sensitivity to temperature variation [14]. Although effective, these methods introduce design complexity, additional power consumption, and higher manufacturing costs, limiting their applicability in many systems.

Therefore, this study proposes a method to correct temperature hysteresis easily and effectively using only the existing MEMS sensor configuration, without any additional hardware requirements, to overcome these limitations. The method estimates hysteresis errors from the correlation between the sensor’s measured temperature change rate and output hysteresis, and formulates a mathematical calibration model.

The effectiveness of this calibration method was evaluated by applying the calibration model to an actual sensor and comparing the performance before and after calibration. In addition, to determine whether the proposed model affects other sensor output characteristics, further tests were conducted based on standard MEMS accelerometer performance indicators [15,16]. The analysis confirmed that the proposed calibration model did not negatively impact other performance metrics.

The remainder of this paper is structured as follows. Section 2 discusses the relationship between the temperature change rate measured by the external temperature sensor mounted outside the MEMS sensor cell, and the internal temperature delay error using a heat transfer model. To validate this relationship, a MEMS accelerometer was tested to evaluate its temperature hysteresis characteristics under varying external temperature change rates and temperature gradients. Section 3 defines the temperature hysteresis calibration model based on the analysis results from Section 2. Section 4 applies the proposed calibration model to the MEMS accelerometer, compares temperature hysteresis characteristics before and after calibration, and evaluates performance based on key sensor metrics. Finally, Section 5 presents a post-analysis of the calibration effect using an existing dataset of MEMS sensor performance test, offering a comprehensive evaluation.

## 2. Relationship Between Internal Temperature Gradient and External Temperature Change Rate

**Figure 1:**
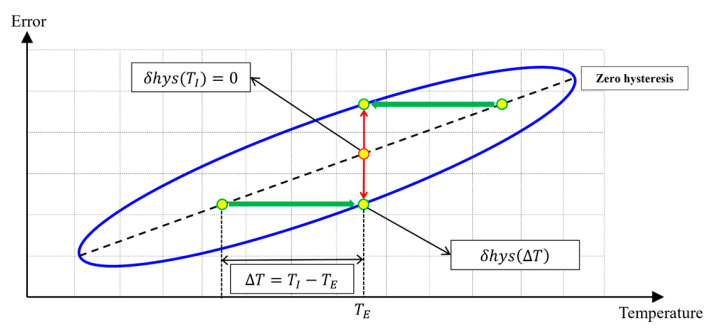
Temperature-hysteresis error of MEMS Sensors.

Although MEMS sensors are designed to be identical, variations in the manufacturing process introduce slight discrepancies, resulting in differences in each sensor’s temperature-dependent output characteristics. These differences lead to unique temperature-dependent output behaviors for each device. In particular, temperature hysteresis arises mainly from internal temperature gradients generated by dynamic external thermal conditions. This hysteresis effect is primarily caused by thermal delay. As illustrated in Figure 1, the sensor’s output is influenced by the temperature delay ΔT, which is defined as the difference between the external temperature $T_{E}$, measured outside the MEMS cell, and the internal temperature $T_{I}$, measured within the MEMS sensor cell. This temperature difference leads to hysteresis in the output signal, denoted by $\delta hys\left(\Delta T\right)$.

Accordingly, this study begins by analyzing the relationship between the internal temperature gradient and the rate of external temperature change, considering the three fundamental heat transfer mechanisms: conduction, convection, and radiation. However, the temperature variation caused by internal heat sources such as structural resonance within MEMS elements are typically on the order to a few to several tens of millikelvins. These are negligible compared to the temperature variations induced by external environmental changes and are thus excluded from the mathematical formulations [17,18].

### 2.1. Transient Heat Transfer Characteristics of MEMS Sensors

The heat transfer in the MEMS devices can be described by the heat balance equation shown in (1), which considers three dominant mechanisms [19]:

1. Heat conduction model, incorporating the thermal conductivity k of materials such as silicon, metal oxides, and other MEMS structural or packaging materials including wires and PCB substrates attached to the MEMS cell;
2. Convection model, governed by Newton’s law of cooling, considering the convective heat transfer coefficient h, which may be affected by residual gases remaining from the fabrication process;
3. Thermal radiation modeled using the Stefan-Boltzmann law.

(1) $$\frac{\partial T_{I}}{\partial t}=\frac{1}{\rho C_{p}}\left(k\nabla^{2}T_{I}\right)-\frac{hA}{\rho C_{p}}\left(T_{I}-T_{E}\right)+\frac{\sigma \varepsilon A}{\rho C_{p}}(T_{E}^{4}-T_{I}^{4})$$

Here, $T_{I}$ and $T_{E}$ represents the internal and external temperatures of the MEMS sensor cell, respectively. The parameter ρ denotes the density, $C_{p}$ is the specific heat capacity, and $\nabla^{2}T_{I}$ represents the spatial distribution of the three-dimensional temperature field. Additionally, A is the cross-sectional area, σ is the Stefan-Boltzmann constant, and ε is the emissivity.

By rearranging (1), the rate of change in the temperature difference between the internal and external temperatures can be expressed as (2). Based on this, the temporal variation in the internal temperature gradient is formulated in (3).

(2) $$\frac{\partial \Delta T}{\partial t}=\frac{\partial T_{I}}{\partial t}-\frac{\partial T_{E}}{\partial t}$$

(3) $$\frac{\partial \Delta T}{\partial t}=\frac{1}{\rho C_{p}}\left(k\nabla^{2}T_{I}-hA\Delta T+\sigma \varepsilon A\left({T_{E}}^{4}-{\left(T_{E}+\Delta T\right)}^{4}\right)\right)-\frac{\partial T_{E}}{\partial t}$$

Considering the characteristics of MEMS devices, where both the density (ρ) and specific heat capacity ($C_{p}$) are very low, heat transfer induced by typical external temperature variations can be regarded as quasi-steady. Therefore, the internal temperature field can be assumed to be spatially uniform ($\nabla^{2}T_{I}\approx 0$), allowing the heat conduction term to be neglected. Under steady-state conditions where ΔT≈0, and during nonlinear temperature external temperature changes such that $\partial T_{E}/\partial t>0$, all terms in (4) can be neglected except for the one associated with the external temperature change rate. Consequently, the internal temperature gradient is proportional to the external temperature change rate.

(4) $$\frac{\partial \Delta T}{\partial t}\approx -\frac{\partial T_{E}}{\partial t}$$

### 2.2. Correlation of Temperature Hysteresis with External Temperature Change Rate

**Figure 2:**
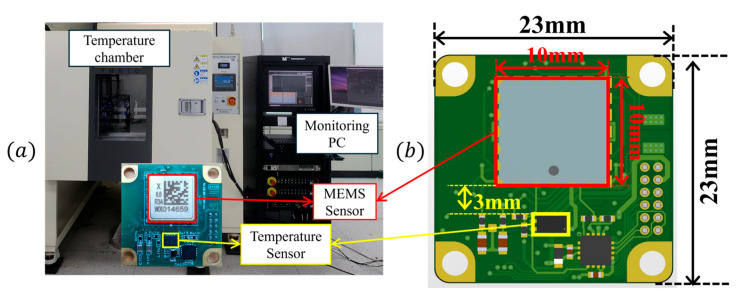
Temperature cycling test environment: (**a**) test environment, (**b**) sensor board.

To verify that the temporal gradient of the internal temperature is proportional to the external temperature change rate, experiments were performed using a self-developed MEMS resonant accelerometer. The test environment is shown in Figure 2a. Three accelerometers were used, and temperature was measured with a sensor [20] mounted on the board approximately 3 mm from the MEMS devices, as depicted in Figure 2b.

**Figure 3:**
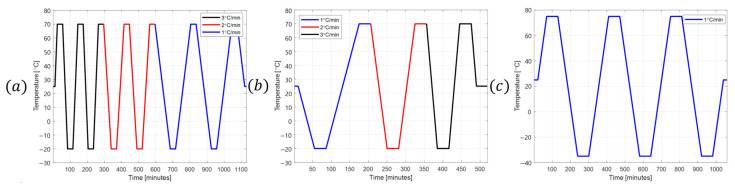
Temperature cycling profile: (**a**) profile #1, (**b**) profile #2, (**c**) profile #3.

Three temperature cycling profiles were applied, each with a change rate of ±3 °C/min over the range −35 °C to +75 °C, as shown in Figure 3. These profiles represent different transient thermal conditions.

**Figure 4:**
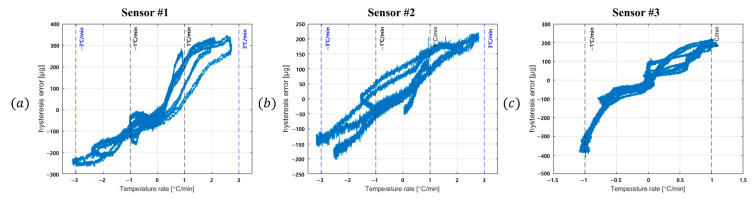
Temperature cycling test result showing the relationship between hysteresis error and external temperature change rate: (**a**) sensor #1, (**b**) sensor #2, (**c**) sensor #3.

For each sensor, the relationship between hysteresis error and the external temperature change rate was obtained and is shown in Figure 4. Figure 4a–c present the correlation between hysteresis error and external temperature change rate for the three sensors. The hysteresis error was calculated by fitting the temperature–output data with a third-order polynomial and subtracting the fitted model from the measured signal.

As shown in Figure 4a–c, the hysteresis error increases with the external temperature change rate in all three sensors. This confirms that the external temperature change rate is the dominant factor influencing temperature hysteresis, compared with internal heat sources or other nonlinear effects.

## 3. Hysteresis Calibration Model

To define a calibration model for temperature hysteresis based on the external temperature change rate, the sensor output error as a function of temperature must first be characterized. The temperature hysteresis, denoted as $hys\left(t,T_{E}\right),$ is calculated from temperature cycling tests conducted at specific temperature change rates and operating temperature ranges, according to the requirements of the application. Based on this data, the sensor output variation with respect to temperature is modeled using an Nth-order polynomial regression with coefficients $p=[p_{1}\;p_{2}\;\cdots p_{N}\;p_{N+1}]\in R^{N+1}$. The hysteresis error as a function of temperature $T_{E}$ is then derived by differentiation, as follows:

(5) $$hys\left(t,T_{E}\right)=Acc\left(t,T_{E}\right)-\left(p_{1}{\left(T_{E}\right)}^{N}+p_{2}{\left(T_{E}\right)}^{N-1}+\cdots +p_{N}{\left(T_{E}\right)}^{1}+p_{N+1}\right).$$

From (5), the Nth-order polynomial regression coefficients for modeling the temperature hysteresis with respect to the external temperature change rate, $(\partial T_{E})/\partial t$, are denoted as $k=[k_{1}\;k_{2}\;\cdots k_{N}\;k_{N+1}]\in R^{N+1}$. These coefficients are estimated using the least squares method, as follows:

(6) $$k={\left(A^{T}\cdot A\right)}^{-1}A^{T}\delta hys\left(t,T_{E}\right),\;A=\left[1\frac{\partial T_{E}}{\partial t}\;{\frac{\partial T_{E}}{\partial t}}^{2}\;\cdots {\frac{\partial T_{E}}{\partial t}}^{N}\right]\in R^{N+1}.$$

As a result, the real-time calibration model derived from (6) is given by (7).

(7) $$\delta hys_{cal}\left(t,\frac{\partial T_{E}}{\partial t}\right)=k_{1}{\left(\frac{\partial T_{E}}{\partial t}\right)}^{N}+k_{2}{\left(\frac{\partial T_{E}}{\partial t}\right)}^{N-1}+\cdots +k_{N}\frac{\partial T_{E}}{\partial t}+k_{N+1}.$$

**Figure 5:**
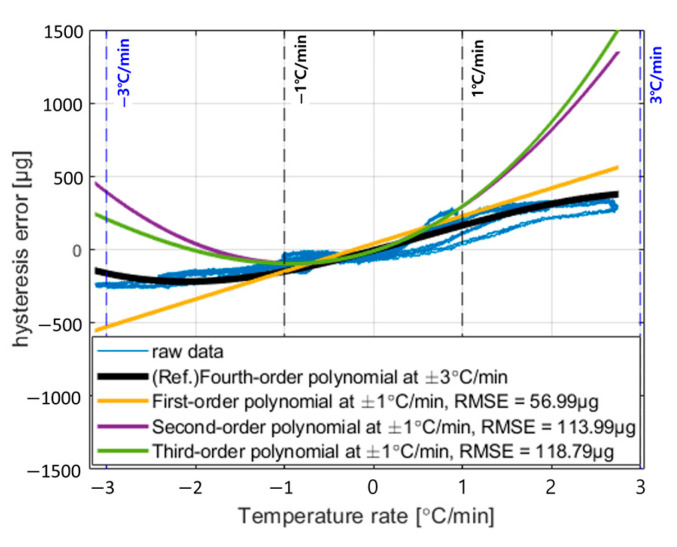
Fitting result of temperature hysteresis error based on temperature rate according to temperature calibration coefficients.

Using a polynomial of second order or higher allows the calibration to capture nonlinear effects as well as errors directly caused by the external temperature change rate. However, if the range of change rates used in the calibration test is narrower than those encountered in operation, calibration accuracy may degrade at higher orders. To illustrate this, the hysteresis error was modeled using first-, second-, and third-order polynomial regressions based on the ±1 °C/min range in Figure 4a–c. The results are shown in Figure 5.

In Figure 5, assuming that the fitting result using a fourth-order polynomial over the entire temperature change rate range represents the true value, the RMSE of the fitted values for first- to third-order polynomials within the ±1 °C/min range are 56.9936 μg, 113.9952 μg, and 118.7971 μg, respectively. This indicates that beyond the calibrated temperature change rate range, the hysteresis calibration performance may deteriorate as the polynomial order increases. Therefore, the calibration model should be defined appropriately to meet the navigation requirements.

**Figure 6:**
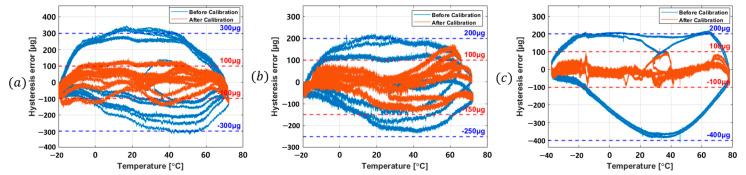
Hysteresis error characteristics before and after calibration: (**a**) Sensor #1, (**b**) Sensor #2, (**c**) Sensor #3.

Finally, using the temperature hysteresis calibration model defined in (7), the temperature hysteresis characteristics of the three MEMS accelerometers analyzed in Section 2 were evaluated and illustrated in Figure 6, showing results before and after applying the third-order calibration coefficients.

Across the entire temperature range, the maximum hysteresis error of Sensor #1 decreased from 348.82 μg to 148.53 μg, Sensor #2 decreased from 224.43 μg to 174.47 μg, and Sensor #3 from 219.46 μg to 105.94 μg. These results indicate that the temperature hysteresis characteristics improved by 57.42%, 22.26%, and 51.73%, respectively, confirming the effectiveness of the calibration model.

## 4. Performance Test Results After Temperature Hysteresis Calibration

**Figure 7:**
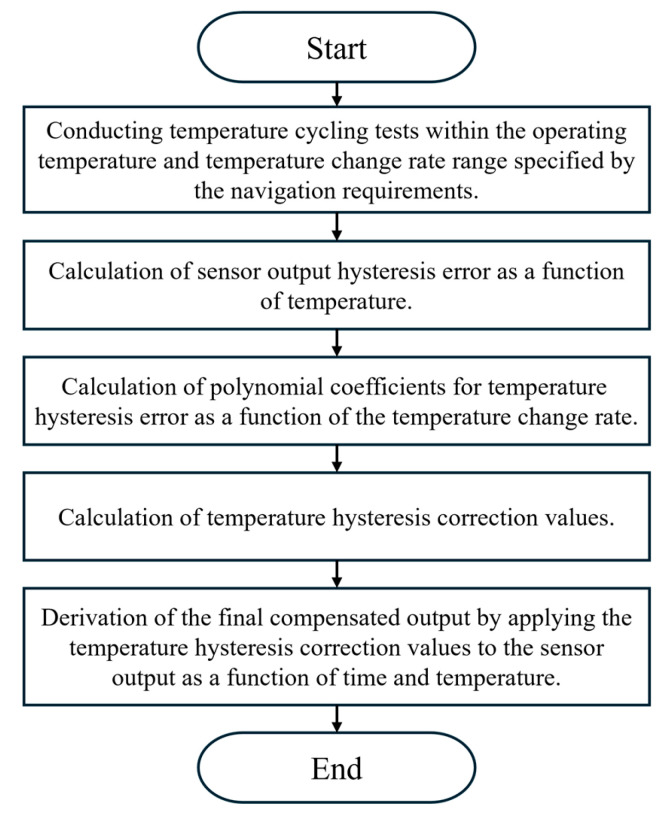
Temperature-hysteresis calibration flowchart.

To evaluate whether the temperature hysteresis calibration model based on the external temperature change rate influences other sensor performance parameters, additional tests were conducted. In Section 4, Sensors 4–6 refer to a different set of devices from Sensors 1–3 used in Section 2 and Section 3. While Sensors 1–3 were employed to analyze the correlation between hysteresis error and the external temperature change rate, Sensors 4–6 were tested independently to validate the calibration model under performance evaluation conditions. The new numbering is introduced to clearly distinguish between these two sets of experiments. The metrics considered were: hysteresis error, bias repeatability, scale factor repeatability, and Allan variance. For this evaluation, three MEMS resonant accelerometers [16] without any prior calibration were selected, ensuring that their maximum hysteresis error over the entire temperature range remained within 500 μg. The calibration procedure was carried out according to the flowchart shown in Figure 7, and the corresponding calibration coefficients were derived.

Performance tests were then conducted to evaluate these metrics before and after applying the calculated temperature hysteresis calibration coefficients. The temperature hysteresis error was measured using the temperature cycling profile shown in Figure 3c under the environmental conditions illustrated in Figure 2, and was defined as the maximum hysteresis error over the entire temperature range.

**Figure 8:**
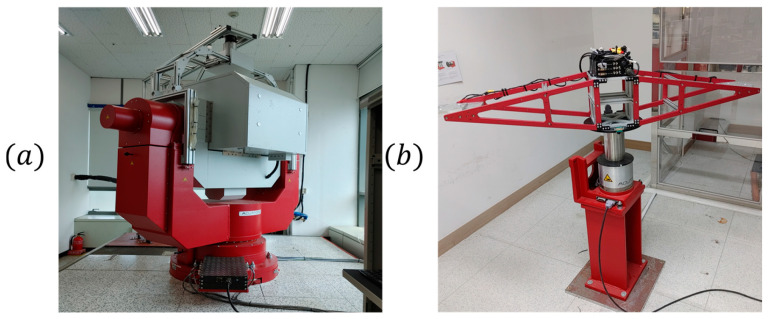
Performance test environment: (**a**) 3-axis rate table [21], (**b**) single-axis rate table [22].

Bias and scale factor repeatability were evaluated using a three-axis rate table system (AC1120s) [21] manufactured by Acutronic Switzerland Ltd. (Rosengartenstrasse 25 8608 Bubikon, Switzerland), as shown in Figure 8a. The test was conducted at −32 °C, +20 °C, and +68 °C by applying ±1 g in a cyclic manner, over five repetitions. The mean RMSE of the value of bias and scale factor for the applied acceleration was then calculated.

For operational range evaluation, a single-axis rate table system (AC3367) [22], also from Acutronic Switzerland Ltd., was used, as shown in Figure 8b. The sensor was subjected to acceleration increments of 10 g from the minimum to the maximum acceleration. Sensor output was measured, and the nonlinearity error of the acceleration output at each input level calculated and expressed in ppm.

An analysis of the performance results presented in Table 1 confirmed that temperature hysteresis calibration effectively reduced the temperature hysteresis error by up to 63%. Bias and scale factor repeatability errors also appeared to improve after calibration compared to before. However, this improvement may have been influenced by the short stabilization time required for thermal equilibrium within each temperature range, which could have introduced initial internal temperature gradients in the sensors. In terms of the operational range, no clear correlation was observed between the nonlinearity error and the temperature change rate before and after calibration. However, in the in-run test, the VRW measured using Allan variance exhibited a slight degradation in performance after calibration. This was likely due to the noise characteristics associated with the temperature change rate, which can affect the sensor output when the calibration model is applied. Nevertheless, the measurement results indicated that the VRW error remained below 1 μg/√Hz, suggesting that the sensor’s performance still meets most operational requirements [23,24,25,26]. Therefore, the observed overall performance improvements can be primarily attributed to the effectiveness of the temperature hysteresis calibration.

In addition to this three-sensor validation, a larger dataset of 48 MEMS accelerometers was also analyzed to assess the broader applicability of the proposed calibration method. To facilitate comparison, these sensors were subsequently classified into two groups according to their maximum pre-calibration hysteresis error: Type 1 (≤500 μg) and Type 2 (>500 μg). This classification was then used in Section 5 to evaluate the average calibration performance across different categories of sensors, as summarized in Table 2.

## 5. Conclusions

This paper presented a calibration method that compensates for temperature hysteresis in MEMS accelerometers using only the existing sensor configuration, without additional hardware. The method estimates hysteresis errors based on the correlation between the external temperature change rate and sensor output, and applies a polynomial regression model for real-time correction.

The proposed calibration was validated through a large-scale evaluation of 48 MEMS accelerometers, as summarized in Table 2. Of these, 32 sensors (Type 1) exhibited pre-calibration hysteresis errors below 500 μg, and 16 sensors (Type 2) exceeded 500 μg. After applying the calibration, Type 1 sensors showed an average improvement of about 42%, while Type 2 sensors improved by about 44%. These results demonstrate that the method is effective across different categories of sensors and hysteresis levels.

Additional evaluations demonstrated that bias and scale factor repeatability improved slightly after calibration, partly due to stabilization dynamics at thermal steps. No clear correlation was found between nonlinearity error and the calibration. In contrast, in-run tests showed minor degradation in velocity random walk and bias instability due to noise associated with the external temperature change rate. However, these errors remained small compared with the overall gains from hysteresis correction, with VRW maintained below 1 μg/√Hz.

In summary, the proposed approach provides a practical and efficient solution for improving the temperature hysteresis performance of MEMS accelerometers, while keeping other performance trade-offs within acceptable limits. This makes it suitable for enhancing the reliability of MEMS sensors in dynamic thermal environments and navigation applications.

## Figures and Tables

**Table 1:** Comparison of result before and after temperature hysteresis calibration for three sensors.

Test No.	List		Operating Temperature	Sensor #4		Sensor #5		Sensor #6	
				Before Calibration	After Calibration	Before Calibration	After Calibration	Before Calibration	After Calibration
1	Temperature Hysteresis [μg]		−35 °C~+75 °C	385.89	254.96	394.81	220.67	453.12	167.02
2	Repeatability	Bias [μg]	−32 °C	118.6	104	20.8	21	27	27.2
			+20 °C	10.5	4.9	12	11.8	3.2	3.1
			+68 °C	63.8	54.6	14.1	13.3	4.6	4.6
		Scale Factor	−32 °C	74.1	73.3	7.3	7.3	15.1	15
			+20 °C	12.5	7.1	10.2	10.2	3.6	3.4
			+68 °C	44.8	39.1	2.7	3.1	2.1	1.8
3	Operation Range	Nonlinearity [ppm]	+25 °C	681.1	687.69	567.12	567.89	252.89	254.04
4	Inrun	VRW [μg√Hz]	+25 °C	7.834	7.936	5.63	5.85	5.38	6.72
		Bias Instability [μg]	+25 °C	0.629	0.636	1.61	1.65	1.07	1.17

**Table 2:** Comparison of results before and after temperature hysteresis calibration using sensor performance test dataset.

Test No.	List		Operating Temperature	Type 1 (≤500 μg, 32 Sensors)			Type 2 (≥500 μg, 16 Sensors)		
				Before Calibration Mean (1σ)	After Calibration Mean (1σ)	Improvement Rate [%]	After Calibration Mean (1σ)	Before Calibration Mean (1σ)	Improvement Rate [%]
1	Temperature Hysteresis [μg]		−35 °C~+75 °C	393.0 (68.2)	226.3 (60.7)	42.4	644.9 (118.6)	357.2 (109.8)	44.6
2	Repeatability	Bias [μg]	−32 °C	41.3 (39.5)	40.7 (37.2)	1.5	46.1 (23.3)	45.4 (23.1)	0.9
			+20 °C	10.8 (8.0)	10.7 (8.3)	0.6	15.7 (13.7)	15.6 (13.7)	0.7
			+68 °C	32.0 (32.1)	31.6 (32.3)	1.3	24.5 (25.4)	23.8 (25.1)	3.1
		Scale Factor	−32 °C	13.1 (23.5)	13.0 (23.0)	0.8	12.4 (11.9)	13.5 (11.9)	−8.8
			+20 °C	4.6 (3.4)	4.2 (2.9)	7.7	3.5 (2.2)	3.4 (2.2)	1.0
			+68 °C	7.1 (11.6)	7.0 (10.3)	1.7	3.8 (1.8)	4.0 (1.9)	−5.7
3	Operation Range	Nonlinearity [ppm]	+25 °C	417.2 (203.4)	418.0 (204.6)	−0.2	373.1 (164.6)	372.5 (164.2)	0.2
4	Inrun	VRW [μg√Hz]	+25 °C	6.1 (1.6)	6.3 (1.6)	−2.5	6.9 (1.9)	7.3 (1.9)	−5.1
		Bias Instability [μg]	+25 °C	1.5 (0.5)	1.6 (0.5)	−2.6	1.6 (0.4)	1.8 (0.5)	−11.3

## Data Availability

Data are contained within the article.

## Abbreviations

The following abbreviations are used in this manuscript:

MEMS	Micro-electromechanical system
RMSE	Root Mean Square Error
VRW	Velocity Random Walk
